# Effects of a Multicomponent Periodized Program in Kinematic and Muscle Activity Characteristics Related to Anterior Cruciate Ligament Injury Mechanism in College Football Players—A Controlled Trial

**DOI:** 10.3390/jfmk10040412

**Published:** 2025-10-21

**Authors:** Loreto Ferrández-Laliena, Lucía Vicente-Pina, Rocío Sánchez-Rodríguez, Graham J. Chapman, Badis Soussi, César Hidalgo-García, María Orosia Lucha-López, José Miguel Tricás-Moreno, Mira Ambrus

**Affiliations:** 1 Unidad de Investigación en Fisioterapia, Spin off Centro Clínico OMT-E Fisioterapia SLP, Universidad de Zaragoza, Domingo Miral s/n, 50009 Zaragoza, Spain; lferrandez@unizar.es (L.F.-L.); l.vicente@unizar.es (L.V.-P.); r.sanchez@unizar.es (R.S.-R.); jmtricas@unizar.es (J.M.T.-M.); 2 Allied Health Research Unit, School of Health, Social Work and Sport, University of Lancashire, Preston PR1 2HE, UK; gchapman2@lancashire.ac.uk; 3 Research Center for Sport Physiology, Hungarian University of Sports Sciences, 1123 Budapest, Hungary; badis.delrio@gmail.com (B.S.); ambrus.mira@tf.hu (M.A.); 4 Department of Sports Medicine and Digital Health Sciences, Faculty of Health and Sport Sciences, Széchenyi István University, 9026 Győr, Hungary

**Keywords:** anterior cruciate ligament, biomechanics, electromyography, angular velocity, female football

## Abstract

**Background:** Given the persistent sex-based disparities in anterior cruciate ligament (ACL) injury prevalence and the heightened susceptibility observed during critical stages of development in female academy-level players, it is necessary to enhance the effectiveness of prevention programs, particularly during change of direction (COD). **Objectives**: This study aims to evaluate whether a multicomponent periodized program modifies three-dimensional knee angular velocity and hamstrings and quadriceps muscle activity during a COD task in under-16 female football players. A secondary objective was to determine whether adaptations differed based on lower limb dominance. **Methods**: A non-randomized, multicenter controlled trial with a pre–post design was conducted involving 35 players (age: 15.50 ± 1.22), allocated to either an intervention (*n* = 17) or control (*n* = 18) group. The intervention group undertook a 12-week multicomponent periodized program within their usual training program whilst the control group undertook their usual training program. The peak and range of thigh and shank angular velocity across three planes, along with the average rectified and peak envelope EMG signals of the Biceps Femoris (BF), Semitendinosus (ST), Vastus Medialis (VM) and Vastus Lateralis (VL), were recorded during the preparation and load phases, using the Change of Direction and Acceleration Test. Three-factor mixed model ANOVAs and non-parametric tests were applied, with statistical significance set at *p* < 0.05. **Results**: Post-intervention analysis revealed significant improvements in sagittal and coronal planes shank angular velocities and thigh coronal and transverse plane angular velocities. Muscle activity patterns also improved, particularly in the ST and VM, suggesting enhanced medial stabilization and neuromuscular control. Functional improvements were most evident in the dominant limb. **Conclusions**: The 12-week multicomponent periodized program effectively modified three-dimensional knee kinematics and muscle activity during a COD task in under-16 female football players.

## 1. Introduction

The anterior cruciate ligament (ACL) remains one of the most severe injuries in female football, due to its injury burden, combining incidence and severity, along with long-term consequences upon return to preinjury sport level [1,2,3]. Although injury prevention strategies implemented reduce knee injury incidence, knee injuries in female football remain the most prevalent compared to other joint locations and are more frequent than in males, especially ACL injury [1,4,5]. At the academy level, ACL injury incidence tends to rise with age and competitive intensity, with two critical peaks identified during the developmental stages of players [2,3,6]. The first occurrence is around the under-16 stage, when the injury incidence reaches 1.7 injuries per 1000 h of exposure, representing the highest rate observed during all academy phases [2]. This increase is associated with peak growth rate and post-pubertal changes, which may reduce motor competence, neuromuscular control, and consequently elevate susceptibility to non-contact injuries [2,3,7]. The second peak emerges during the under-19 to under-23 stages, coinciding with the transition to the elite-level period, reaching 1.1 injuries per 1000 h of exposure. During this stage, players are exposed to intensified physical demands and competitive pressure, while often still undergoing physical maturation [2,8].

Epidemiological studies indicate that approximately 59–64% of ACL injuries occur via a non-contact mechanism, particularly in females. Change of Direction (COD) has been identified as the most prevalent task associated with injury mechanism, reported in 48–70% cases, due to the involvement of multiplanar knee kinematics [9,10,11]. Football matches frequently involve COD actions, often executed in a high-pressure sports context [12]. In these high-intensity scenarios, functional demands may exceed the tensile capacity of the ACL, promoting injury, particularly when players use their dominant limb (DL) for stabilization under competitive pressure [13,14,15]. ACL injuries frequently occur in unpredictable defensive duels, accounting for approximately 60–73% of sport-specific scenarios. In this context, limited reaction time constrains the DL to adopt an unaccustomed stabilizing role, elevating injury susceptibility [9,15,16]. Clinical guidelines support the implementation of prevention programs, which have been shown to reduce non-contact injuries by 67% and ACL injuries by 53% [17,18]. These programs aim to minimize potentially dysfunctional adjustments by modifying movement patterns and addressing neuromuscular deficits [19,20]. Based on clinical recommendations, such programs should adopt a multicomponent structure and be guided by biomechanically informed progression criteria within a periodized model, involving a sequential increase in functional demands [17,21]. Furthermore, evidence suggests greater efficacy when applied to academy-level players, as neuromuscular plasticity during development facilitates motor pattern adaptation more efficiently than in elite players with automatized motion patterns [17,18].

The effectiveness of prevention programs depends on evidence-based design from risk factor analysis studies. However, most reported functional improvements have been limited to landing tasks [19,20,22]. No significant functional changes have been observed during COD tests, likely due to the multiplanar knee kinematics and rapid adjustments involved. This complexity makes it difficult to capture these adaptations using conventional kinematic variables, such as joint range of motion [12,21]. Consequently, angular velocity has been proposed as an alternative parameter for characterizing joint movement quality, as it reflects both the speed and direction of joint angular excursion, as evidenced by segmental motion directly regulated by muscle activity, closely linked to motor control [23,24,25]. Valgus collapse is the primary kinematic mechanism of ACL rupture, involving a combination of multiplanar kinematics [9,11,26,27]. Most valgus collapse kinematics are oriented in the sagittal plane, where limited knee flexion increases anterior tibial translation, increasing ACL strain [11,28,29,30,31]. Simultaneously, pivot-shift mechanics in the coronal and transverse planes, marked by hip adduction, knee abduction, and tibial rotation, further increase medial tibial translation and ACL tensile capability [11,32,33]. Electromyographic (EMG) muscle activity data support that altered neuromuscular motor control increases injury risk [17,18,34]. The hamstrings, particularly the semitendinosus (ST), act as main synergists of the ACL by inducing posterior tibial translation, limiting tibial rotation, and compressing the medial knee compartment [28,35,36]. In contrast, excessive quadriceps activation at low flexion angles promotes anterior tibial translation, heightening ACL stress [34]. Female players at risk of ACL injury often exhibit quadriceps dominance accompanied by imbalanced lateral-to-medial thigh muscle activity, characterized by preferential muscle function of the Biceps Femoris (BF) and Vastus Lateralis (VL) over the ST and Vastus Medialis (VM) [25]. This lateral dominance may increase compressive forces on the lateral compartment, exacerbating tibial rotation and promoting medial compartment gapping through a pivot-shift mechanism [25,36,37,38]. Combined, these multiplanar mechanisms may exceed the structural tolerance of the ACL, leading to rupture without external contact [11,39].

Given the persistent sex-based disparities in ACL injury prevalence and the heightened susceptibility observed during critical stages of development in female academy-level players, it is necessary to improve the effectiveness of prevention programs, particularly in COD functional improvements, as the most common ACL injury mechanism [1,2]. Addressing recent literature key gaps, this study proposes the implementation of a multicomponent periodized program based on neuromuscular training, COD technique modification, and real-time feedback [17,19,21]. This comprehensive approach aims to enhance movement quality and motor control in female football players, with particular focus on the under-16 academy stage, where neuromuscular adaptations are still highly modifiable [2,3]. Therefore, our main objective was to evaluate whether a multicomponent periodized program modifies three-dimensional knee angular velocities and hamstrings and quadriceps muscle activity during a COD task in female football players. It was hypothesized that implementing a multicomponent periodized program would lead to improved movement quality, assessed by angular velocity, and motor control, evaluated by muscle activity analysis. Moreover, a secondary objective was to assess whether the effects of the training program on movement quality and motor control differ between the DL and NDL.

## 2. Materials and Methods

### 2.1. Study Design

A non-randomized, multicenter controlled trial with a repeated-measures pre- to post-test design was conducted. Female under-16 stage football players from Újpest Football Club and Honvéd Football Club from Budapest, Hungary, and Ebro Sport Club and Valdefierro Sport Club from Zaragoza, Spain were recruited. Players from Újpest Football Club were assigned to the intervention group, which completed a 12-week intervention program replacing their regular team warm-up, following clinical guidelines recommendations [17]. The control group was composed of players who met all inclusion criteria from the remaining participating teams. The study was approved by the Research Ethics Committee of the Community of Aragón (code PI20/127) and conducted in accordance with the ethical principles of the Declaration of Helsinki [40]. The study design followed CONSORT, CERT, and TIDieR reporting guidelines [41].

### 2.2. Participants

Participant recruitment was coordinated by the University of Zaragoza (Spain) and the Hungarian University of Sports Science (Hungary), who contacted the club presidents to establish collaboration agreements. All players were required to have competed during the 2023/24 season in a female under-16 regional league and training four days per week, with each session lasting 90 min. Players from both groups were required to attend all scheduled training sessions during the 12-week period. Participants who sustained injuries that prevented regular participation or failed to comply with the intervention protocol or standard training schedule were excluded from the final analysis to ensure comparable exposure to physical load at the post-intervention assessment [21,42]. Goalkeepers were not included in this investigation [12]. Informed consent was obtained from each player and their parents or legal guardians prior to final inclusion. A total of 85 players from the recruited teams met the eligibility criteria. Each eligible player was assigned a number, and participants were randomly selected into the study using a random number generator until 19 were assigned to the intervention group and 19 to the control group. Throughout the follow-up period, the control group continued performing their standard field-based warm-up routines.

### 2.3. Procedure

**Figure 1:**
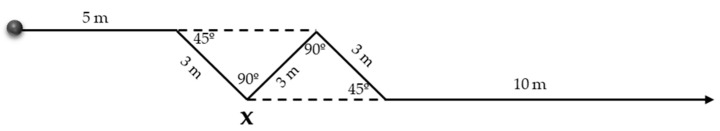
CODAT. The starting position is indicated with the dark circle, and the cross identifies the change of direction task that was recorded.

Participants took part in a single pre-intervention and a single post-intervention testing session, during which thigh and shank kinematics, as well as hamstrings and quadriceps muscle activity, were recorded. Prior to each session, they completed a standardized 10 min warm-up consisting of mobility exercises and variable-intensity running with COD drills [12]. Each participant then completed six trials of the Change of Direction and Acceleration Test (CODAT), three using the DL and three using the non-dominant limb (NDL), all performed at maximum effort, with 45 s of recovery between trials [35]. The CODAT was selected based on prior research indicating that 90° directional changes impose a greater demanding challenge for the players, providing a more effective assessment of knee stability [43,44]. This test combines sprinting with stabilization and acceleration demands, incorporating four COD, two at 45° and two at 90°, interspersed with 3 m sprints and a final 10 m sprint. The second 90° COD was selected for data analysis, following previous recommendations (see Figure 1) [43]. Participants completed a familiarization period to minimize learning bias. Each participant was assigned a unique anonymized code to reduce potential analysis bias, thereby ensuring the reliability and objectivity of the data collected during the pre- and post-intervention testing sessions [43].

### 2.4. Sensor Placement

Surface EMG signals were recorded using four Trigno Avanti wireless sensors (Delsys Inc., Natick, Massachusetts, USA), sampled at 1000 Hz, and placed over the BF, ST, VM, and VL muscles, in accordance with SENIAM guidelines [45,46]. Throughout data collection, the signal-to-noise ratio was regularly monitored to ensure the quality of EMG recordings.

An additional Trigno Avanti sensor was placed on the anterior tibial tuberosity to capture shank angular velocities, whilst the VL sensor was used to capture thigh angular velocities [47,48]. These sensors include integrated inertial measurement units (IMUs), which obtain angular velocity data captured via their built-in triaxial gyroscope [47,48].

### 2.5. Intervention Period—12-Week Multicomponent Periodized Program

A 12-week multicomponent periodized program, based on neuromuscular training, COD technique modification, and real-time feedback (Appendix A), was implemented in the intervention group. The program was carried out on an artificial grass football pitch at the Újpest Football Club Sports Center, in Budapest, and supervised by an advanced-level coach, recognized by the Union of European Football Associations, UEFA A-licensed, and a physiotherapist specialized in biomechanics. The training schedule was structured into three 30 min sessions per week, replacing the regular warm-up at the beginning of designated training days [17]. Sessions were held on Match-5, Match-4, and Match-2 days, referring to the number of days before the match, in accordance with load management principles within a structured microcycle to avoid overload on match day [49]. Each session was structured into two segments: the initial 10 min warm-up focused on aerobic mobility and neuromuscular activation, followed by a 20 min main section [17]. The main section consisted of two rounds of a six-exercise circuit specifically designed to enhance COD performance, as detailed in Appendix A.

Players worked in pairs, facilitating continuous peer feedback [50]. Each exercise was performed for one minute, resting for 30 s between stations. Coaches provided real-time internal feedback and recorded the drills to offer self-visual feedback after training [21,50]. The program was structured into three monthly mesocycles, each progressively increasing in difficulty and aligned with specific performance objectives [51,52]. The structured progression for each mesocycle is detailed in Appendix A.1 (Phase 1), Appendix A.2 (Phase 2), and Appendix A.3 (Phase 3). The first phase targeted neuromuscular and proprioceptive training focused on activating the synergist muscles of the ACL to enhance the valgus collapse dysfunctional pattern, using bodyweight exercises [18]. The second phase emphasized correcting and automating functional movement patterns during different amplitudes COD task [12,21]. In the final phase, a sport-specific context was introduced through dual-task drills involving small-sided attack-defense scenarios and ball integration, ensuring that each task had both a functional and tactical objective [53]. During each phase, the same exercises were performed in every session to promote learning and the automation of motor patterns [51].

The control group completed 12 weeks of their standard physical training routines on an artificial grass football pitch. To ensure comparability of training exposure between groups, external and internal load, adherence, and competition demands, based on the number of matches played, were systematically standardized and monitored. External load was monitored through session volume (90 min per session), frequency (four sessions per week), and intensity using the Rating of Perceived Exertion (RPE) as an indicator of compliance with planned performance demands [54,55,56]. Target RPE values were set at 8–9 for the initial sessions and 6–7 for the final sessions of each microcycle [57]. After each session, the session duration was recorded by the team coach, and the players self-reported RPE using a self-designed Google Forms questionnaire. Weekly training load was verified by comparing recorded values against pre-planned targets and quantified as the product of these three components to ensure comparability across groups [54,55,56]. Internal load was tracked using a standardized wellness questionnaire (1–7 scale) assessing sleep quality, stress, muscle soreness, and perceived fatigue, thereby capturing individual tolerance and adequacy of the training stimulus throughout the intervention [58].

### 2.6. Data Analysis

EMG and angular velocity data were exported to C3D format and processed using Visual3D software v2024.08.3 (HAS Motion, Kingston, ON, Canada). EMG signals were filtered using a second-order Butterworth high-pass filter with a cut-off frequency of 40 Hz to minimize movement artifacts, then full-wave rectified, and low-pass filtered with a 15Hz cut-off frequency [59]. The maximum observed signal from the filtered data across all trials and muscles was used to normalize the muscle activity during the preparation (PREP) phase, defined as 100ms prior to ground contact to the frame immediately before initial contact, and the loading (LOAD) phase, defined as initial contact to final foot ground contact [39,60]. Accordingly, average and peak muscle activity outcomes were reported for analysis during both PREP and LOAD phases, expressed as a percentage of maximum.

The maximums, minimums, and ROM angular velocities in the sagittal, coronal, and transverse planes were extracted for analysis during the LOAD phase [47]. The maximum values correspond to the highest positive values for each plane: flexion in sagittal, varus in coronal, and external rotation in the transverse plane. In contrast, the minimum values correspond to the lowest negative values for each plane, extension in sagittal, valgus in coronal, and internal rotation in the transverse plane. The ROM angular velocity was defined as the difference between the maximum and minimum angular velocities for each plane, respectively. All angular velocity values were reported in degrees per second (°/s).

### 2.7. Sample Size

The sample size was estimated using the GRANMO 8.0 calculator (IMIM, Barcelona, Spain). ST muscle activity was selected as the primary outcome, based on prior evidence supporting its key role as an ACL synergist during the stabilization process, and therefore as a critical variable expected to show significant improvement following the intervention [25,36,39,42]. Based on the data reported by Zebis et al., a minimum expected difference of 15% in peak ST muscle activity, expressed as EMG maximum capacity, during the PREP phase of a COD test was assumed [36,61]. Considering a two-sided test, an alpha risk of 0.05, and a statistical power of 80%, a minimum of 17 participants per group was required. An estimated dropout rate of 10% was also considered.

### 2.8. Statistical Analysis

Shapiro–Wilk tests were conducted to assess the normality of the data. For normally distributed variables, three-factor mixed model ANOVAs (2 group × 2 time point × 2 limb) were performed to examine differences in kinematic angular velocity and average and peak EMG variables across groups (intervention vs. control), time points (pre- vs. post-intervention), and limb dominance (DL vs. NDL). When significant interactions were found between group and time (Group*Time) and limb and time (Limb*Time), independent t-tests were conducted to compare values between the intervention and control groups or between DL and NDL at both pre- and post-intervention time points. In cases where a three-way interaction was significant (Group*Limb Dominance*Time), separate paired *t*-tests were performed within the intervention and control groups to assess pre- to post-intervention changes for both DL and NDL. For non-normally distributed data, non-parametric tests were used. Mann–Whitney U test was applied to compare the intervention and control groups at pre- and post-intervention time points, with analyses conducted separately for DL and NDL values. The Wilcoxon signed-rank test was performed for within-group (pre- to post-intervention) comparisons of DL and NDL separately in both the intervention and control groups. Statistical significance was set at *p* < 0.05. In addition, effect sizes were calculated using Cohen’s d for significant interactions in normally distributed variables during post hoc pairwise comparisons as well as in all non-normally distributed variables. Effect sizes were interpreted as very large when d > 1.0. All analyses were performed using SPSS Statistics v25.0 (IBM Corp., Armonk, NY, USA).

## 3. Results

A total of 38 players were initially recruited and assigned to the intervention group (*n* = 19) or the control group (*n* = 19). At follow-up, 35 participants completed the study (intervention group, *n* = 17; control group, *n* = 18). One participant in the intervention group was lost to follow-up, and two participants, one from each group, were excluded due to injury sustained during the intervention period. The demographic characteristics of the participants are presented in Table 1. No significant differences were observed in demographic characteristics between groups.

**Table 1:** Demographic characteristics of the players.

		Total (*n* = 35)	Intervention Group (*n* = 17)	Control Group (*n* = 18)
Age (years)		15.50 ± 1.22	15.29 ± 1.16	15.70 ± 1.27
Height (cm)		164.67 ± 6.47	165.96 ± 5.90	163.37 ± 7.05
Weight (Kg)	Pre	58.83 ± 6.08	57.23 ± 4.59	60.43 ± 7.57
	Post	58.46 ± 6.92	56.55 ± 5.90	60.38 ± 7.94
Limb Dominance (Right/Left)		33/2	17/0	16/2
Football Experience (years)		7.47 ± 1.15	7.31 ± 1.14	7.62 ± 1.15

### 3.1. Kinematic Analysis Findings

Table 2 shows the descriptive analysis for normally distributed data, along with results from the three-factor mixed model ANOVA for peak and ROM thigh and shank angular velocities across sagittal, coronal, and transverse planes during the CODAT, performed with the DL and NDL. No significant main effects or interactions were observed for thigh angular velocity in the coronal or transverse planes (*p* > 0.05). In the sagittal plane, the mixed model ANOVA revealed a significant limb dominance by time interaction (Limb Dominance*Time) for thigh extension angular velocity (*p =* 0.021) (Table 2). Post hoc independent *t*-test analysis revealed that DL exhibited a significant reduction in thigh extension angular velocity from pre- to post-intervention (*p =* 0.040) (Table 7). Additionally, a significant three-way interaction (Group*Limb Dominance*Time) was found for thigh sagittal ROM angular velocity (*p* = 0.025) (Table 2). Post hoc paired *t*-tests, conducted separately for the intervention and control groups, indicated that the NDL in the control group exhibited a significant decrease in thigh sagittal ROM angular velocity from pre- to post-intervention (*p* = 0.015) (Table 8).

Table 3 presents the descriptive statistics for non-normally distributed data, along with results from independent comparisons between groups and paired comparisons within groups, for peak and ROM angular velocities of the thigh and shank across sagittal, coronal, and transverse planes during the PREP and LOAD phases of CODAT. Data are reported separately for the DL and NDL at pre- and post-intervention time points.

**Table 2:** Mean ± standard deviation (SD) values for normally distributed data are presented, along with results from the three-factor mixed model ANOVA for peak and range of motion (ROM) thigh and shank angular velocities across sagittal, coronal, and transverse planes during the CODAT, performed with the dominant limb (DL) and non-dominant limb (NDL).

	PRE—Intervention				POST—Intervention				*p*-Value		
	Intervention		Control		Intervention		Control				
	DL	NDL	DL	NDL	DL	NDL	DL	NDL			
		*Mean ± Standard Deviation*							Group * Time	Limb Dom * Time	Three-Way Interaction Effect
Variables Angular Velocity (°/s)											
***Sagittal Plane***											
Thigh Extension	−411.48 ± 93.96	−371.94 ± 108.07	−372.98 ± 97.44	−416.20 ± 66.06	−343.09 ± 89.08	−354.41 ± 58.25	−370.26 ± 90.12	−367.02 ± 79.94	0.563	0.021 *	0.100
ROM Thigh Sagittal	619.19 ± 164.63	503.77 ± 136.82	640.71 ± 123.78	802.91 ± 241.33	554.06 ± 128.95	541.26 ± 140.35	651.36 ± 192.79	645.53 ± 241.23	0.320	0.585	0.025 *
***Coronal Plane***											
Thigh Varus/Abd	228.21 ± 136.64	178.56 ± 107.91	260.95 ± 129.68	270.16 ± 125.84	175.06 ± 87.90	190.29 ± 118.18	219.12 ± 102.56	200.84 ± 113.94	0.377	0.581	0.242
ROM Thigh Coronal	434.81 ± 202.76	371.52 ± 167.96	491.34 ± 196.53	486.34 ± 158.10	345.22 ± 114.74	315.97 ± 133.25	417.75 ± 195.54	416.66 ± 183.92	0.987	0.396	0.795
***Transverse Plane***											
Shank Internal Rotation	519.46 ± 155.96	438.30 ± 197.74	512.17 ± 221.36	568.96 ± 271.85	441.06 ± 191.96	372.73 ± 151.55	632.78 ± 300.90	481.96 ± 223.89	0.235	0.460	0.141

* and bold Denotes significance. (DL) Dominant Limb/(NDL) Non-Dominant Limb/(Limb Dom) Limb Dominance/ROM (Range of Motion).

In the sagittal plane, significant post-intervention differences were found between groups in shank extension, flexion, and sagittal ROM angular velocities in both limbs. The intervention group demonstrated higher shank extension (*p* = 0.008 for DL; *p* = 0.019 for NDL), shank flexion (*p =* 0.027 for DL; *p =* 0.011 for NDL), and shank sagittal ROM (*p* = 0.005 for DL; *p* = 0.019 for NDL) angular velocities. Notably, in the pre-intervention assessment, the intervention group showed significantly lower shank flexion angular velocity in NDL compared to the control group (*p* = 0.027). Therefore, the intervention group exhibited a significant increase in shank flexion angular velocity from pre- to post-intervention in NDL (*p* = 0.017). There were no significant differences in thigh flexion angular velocity (Table 3).

In the coronal plane, significant post-intervention differences were found between groups in thigh valgus angular velocity in NDL, as well as in shank varus and coronal ROM angular velocities in DL. The intervention group demonstrated lower thigh valgus (*p* = 0.008) angular velocity in NDL and higher shank varus (*p* = 0.022) and shank coronal ROM (*p* = 0.029) angular velocities in DL. Additionally, a significant pre-intervention difference between groups was observed in shank valgus angular velocity in DL, with the intervention group showing higher shank valgus angular velocity compared to the control (*p* = 0.022); however, this difference was no longer present post-intervention. Finally, the intervention group exhibited a significant increase in shank varus angular velocity from pre- to post-intervention in NDL (*p* = 0.013) (Table 3).

In the transverse plane, no significant post-intervention differences were found between groups. However, the intervention group showed significant pre- to post-intervention changes in thigh internal and external rotation angular velocities in DL, as well as in thigh transverse ROM angular velocity in both limbs. The intervention group demonstrated significant decreases in thigh external and internal rotation (*p* = 0.010 and *p* = 0.017, respectively) in DL, as well as significant decreases in transverse ROM (*p* = 0.002 for DL; *p* = 0.014 for NDL) angular velocities in both limbs. Finally, the control group showed significantly higher shank external rotation (*p* = 0.027) and shank transverse ROM (*p* = 0.042) angular velocities in NDL compared to the intervention group during the pre-intervention assessment. These differences were no longer present post-intervention, although no significant pre- to post-intervention changes were observed in either group (Table 3).

### 3.2. Muscle Activity Analysis Findings

Table 4 and Table 5 show the descriptive analysis for normally distributed data, along with results from the three-factor mixed model ANOVA for average and peak EMG muscle activity, for the PREP and LOAD phases, respectively, of the CODAT, performed with the DL and NDL. No significant main effects or interactions were observed for muscle activity EMG in the PREP phase (*p* > 0.05) (Table 4). In the LOAD phase, the mixed model ANOVA revealed a significant limb dominance by time interaction (Limb Dominance*Time) for peak BF muscle activity (*p =* 0.013) (Table 5). Post hoc independent *t*-test analysis revealed that NDL exhibited a significant reduction in peak BF muscle activity from pre- to post-intervention (*p =* 0.003) (Table 7). Additionally, a significant three-way interaction (Group*Limb Dominance*Time) was found for peak ST muscle activity (*p* = 0.006) (Table 5). Post hoc paired *t*-tests, conducted separately for the intervention and control groups, indicated that the DL in the intervention group exhibited a significant decrease in peak ST muscle activity from pre- to post-intervention (*p* = 0.006) (Table 8).

**Table 3:** Median and interquartile range [Q1–Q3] values for non-normally distributed data are presented along with results from the Mann–Whitney U test between groups and with results from the Wilcoxon test within-group, for peak and range of motion (ROM) thigh and shank angular velocities across sagittal, coronal, and transverse planes during the CODAT, performed with the dominant (DL) and non-dominant limb (NDL).

	PRE—Intervention								POST—Intervention								Wilcoxon *p*-Value (PRE—POST)		Effect Size d Cohen (PRE—POST)	
	Intervention		Control		Mann Whitney U		Effect Size d Cohen		Intervention		Control		Mann Whitney U		Effect Size d Cohen					
	DL	NDL	DL	NDL	DL	NDL	DL	NDL	DL	NDL	DL	NDL	DL	NDL	DL	NDL				
*Median [Interquartile Range [Q1, Q3]*																	Intervention Related Samples Analyses	Control Related Samples Analyses	Intervention Related Samples Analyses	Control Related Samples Analyses
Variables Angular Velocity (°/s)																				
***Sagittal Plane***																				
Thigh Flexion	188.33 [90.69, 323.76]	144.06 [67.54, 175.93]	267.02 [170.31, 360.21]	375.45 [191.97, 575.66]	0.318	0.000 *	−1.62 ^†^	6.69 ^†^	222.17 [146.82, 274.83]	173.98 [78.86, 303.44]	286.35 [138.26, 412.08]	213.20 [139.41, 430.26]	0.173	0.273	2.13 ^†^	2.10 ^†^	DL: 0.653 NDL: 0.124	DL: 0.349 NDL: 0.085	DL: 0.05 NDL: 2.38 ^†^	DL: 0.38 NDL: 2.08 ^†^
Shank Extension	−158.87 [−305.97, −45.26]	−140.63 [−248.83, 30.78]	−66.76 [−150.55, −23.09]	−53.84 [−108.92, 0.17]	0.144	0.403	−1.62 ^†^	1.57 ^†^	−176.44 [−345.17, −102.29]	−153.94 [−304.39, −41.63]	−41.03 [−98.31, 36.92]	3.73 [−129.90, 45.83]	0.008 *	0.019 *	2.60 ^†^	1.42 ^†^	DL: 0.469 NDL: 0.679	DL: 0.396 NDL: 0.215	DL: 0.92 NDL: 0.66	DL: 0.28 NDL: 0.29
Shank Flexion	631.67 [552.42, 762.21]	543.08 [463.61, 714.58]	487.25 [404.33, 677.64]	626.43 [483.73, 727.82]	0.959	0.027 *	3.14 ^†^	1.10 ^†^	617.69 [506.00, 898.83]	666.72 [567.76, 929.40]	512.74 [404.97, 636.21]	483.38 [404.54, 602.36]	0.027 *	0.011 *	3.90 ^†^	2.78 ^†^	DL: 0.836 NDL: 0.017 *	DL: 0.647 NDL: 0.122	DL: 0.42 NDL: 3.04 ^†^	DL: 0.85 NDL: 1.20 ^†^
ROM Shank Sagittal	842.77 [612.61, 1141.90]	698.16 [450.97, 956.79]	546.81 [477.07, 784.80]	643.83 [506.10, 786.20]	0.187	0.695	2.84 ^†^	0.24	784.35 [625.18, 1118.16]	820.66 [567.62, 1232.52]	502.79 [406.37, 698.93]	442.90 [366.29, 768.80]	0.005 *	0.019 *	3.75 ^†^	2.19 ^†^	DL: 0.535 NDL: 0.109	DL: 0.557 NDL: 0.078	DL: 0.76 NDL: 2.08 ^†^	DL: 0.64 NDL: 0.50
***Coronal Plane***																				
Thigh Valgus	−162.31 [−292.84, −130.02]	−167.46 [−245.99, −135.90]	−202.07 [−266.43, −162.50]	−214.72 [−264.86, −162.22]	0.303	0.258	1.03 ^†^	0.98	−153.86 [−216.75, −129.09]	−124.80 [−163.15, −100.45]	−158.56 [−200.94, −115.53]	−197.91 [−259.17, −121.60]	1.000	0.008 *	1.07 ^†^	4.30 ^†^	DL: 0.246 NDL: 0.025 *	DL: 0.122 NDL: 0.711	DL: 1.65 ^†^ NDL: 3.81 ^†^	DL: 1.10 ^†^ NDL: 0.02
Shank Valgus	−387.44 [−466.28, −216.66]	−257.58 [−395.05, −180.16]	−254.53 [−391.80, −157.22]	−287.05 [−411.14, −201.79]	0.022 *	0.695	2.31 ^†^	0.68	−266.01 [−406.42, −184.66]	−174.69 [−319.93, −145.55]	−216.89 [−293.05, −119.38]	−170.27 [−306.65, −91.40]	0.245	0.568	1.25 ^†^	0.25	DL: 0.501 NDL: 0.134	DL: 0.306 NDL: 0.078	DL: 0.94 NDL: 1.19 ^†^	DL: 0.40 NDL: 1.93 ^†^
Shank Varus	389.33 [249.25, 697.08]	245.07 [174.75, 307.50]	256.33 [209.80, 308.46]	255.11 [236.53, 391.75]	0.020 *	0.251	3.70 ^†^	1.64 ^†^	398.97 [273.07, 509.85]	358.59 [230.03, 536.36]	277.35 [144.60, 378.08]	232.08 [180.34, 349.75]	0.022 *	0.096	3.00 ^†^	1.11 ^†^	DL: 0.642 NDL: 0.013 *	DL: 0.913 NDL: 0.372	DL: 0.83 NDL: 3.13 ^†^	DL: 0.03 NDL: 0.32
ROM Shank Coronal	754.27 [468.27, 944.63]	436.92 [374.21, 707.55]	552.00 [390.02, 662.97]	682.15 [456.80, 764.81]	0.030 *	0.211	3.46 ^†^	1.42 ^◊^	592.03 [527.55, 995.34]	544.13 [371.76, 813.52]	425.36 [268.73, 708.48]	409.67 [290.07, 604.89]	0.029 *	0.110	2.26 ^†^	0.81	DL: 0.469 NDL: 0.642	DL: 0.500 NDL: 0.058	DL: 1.01 ^†^ NDL: 1.32 ^†^	DL: 0.24 NDL: 0.68
***Transverse Plane***																				
Thigh Int Rot	−483.39 [−729.41, −293.17]	−355.65 [−615.18, −262.08]	−360.49 [−509.59, −240.07]	−504.46 [−676.22, −396.81]	0.232	0.195	2.29 ^†^	0.87	−279.71 [−478.98, −171.26]	−257.63 [−499.43, −171.70]	−328.95 [−510.58, −234.47]	−385.81 [−527.98, −263.31]	0.424	0.134	0.59	1.80 ^†^	DL: 0.017 * NDL: 0.076	DL: 0.647 NDL: 0.028 *	DL: 3.04 ^†^ NDL: 2.27 ^†^	DL: 0.52 NDL: 2.36 ^†^
Thigh Ext Rot	524.72 [443.35, 799.60]	399.39 [268.46, 524.53]	358.95 [258.58, 506.70]	564.44 [310.06, 614.90]	0.014 *	0.126	2.87 ^†^	1.88 ^†^	373.62 [229.22, 511.09]	359.34 [269.68, 463.43]	285.87 [185.29, 537.21]	406.49 [315.98, 474.26]	0.832	0.386	0.09	0.58	DL: 0.010 * NDL: 0.619	DL: 0.327 NDL: 0.122	DL: 3.92 ^†^ NDL: 0.40	DL: 0.93 NDL: 1.71 ^†^
ROM Thigh Transverse	1100.97 [781.14, 1373.19]	853.41 [593.04, 1063.16]	730.85 [525.73, 1121.73	1012.31 [702.57, 1233.77]	0.959	0.211	2.86^†^	1.68^†^	711.71 [418.30, 946.61]	619.00 [483.08, 909.21]	668.46 [486.33, 981.02]	842.64 [643.48, 971.74]	0.961	0.184	0.35	1.42 ^†^	DL: 0.002 * NDL: 0.014 *	DL: 0.327 NDL: 0.076	DL: 3.85 ^†^ NDL: 1.65 ^†^	DL: 0.87 NDL: 2.35 ^†^
Shank Ext Rot	−382.95 [−673.24, −272.65]	−385.12 [−448.65, −216.54]	−440.65 [−707.45, −230.45]	−611.39 [−683.34, −363.50]	0.959	0.027 *	0.72	2.90 ^†^	−320.57 [−617.07, −234.49]	−402.48 [−578.84, −261.21]	−485.96 [−959.60, −292.64]	−493.12 [−566.04, −273.31]	0.303	0.483	1.76 ^†^	1.23 ^†^	DL: 0.438 NDL: 0.438	DL: 0.472 NDL: 0.420	DL: 0.33 NDL: 0.93	DL: 0.81 NDL: 0.75
ROM Shank Transverse	923.06 [688.65, 1223.11]	900.56 [509.76, 1009.47]	972.71 [605.50, 1323.60]	1090.74 [765.62, 1423.79]	0.959	0.042 *	0.43	2.86 ^†^	803.81 [543.57, 1218.70]	730.85 [650.87, 944.61]	1095.36 [705.99, 1664.09]	973.91 [693.96, 1146.79]	0.096	0.126	2.69 ^†^	2.26 ^†^	DL: 0.469 NDL: 1.000	DL: 0.248 NDL: 0.157	DL: 1.06 ^†^ NDL: 0.28	DL: 1.44 ^†^ NDL: 1.34 ^†^

* and bold Denotes significance/^†^ denotes effect size d > 1. (DL) Dominant Limb/(NDL) Non-Dominant Limb.

**Table 4:** Mean ± standard deviation (SD) values for normally distributed data are reported, along with results from a three-factor mixed model ANOVA for average and peak Biceps Femoris, Semitendinosus, Vastus Medialis, and Vastus Lateralis muscle activity during the preparation phase of the CODAT, performed with the dominant limb (DL) and non-dominant limb (NDL).

	PRE—Intervention				POST—Intervention				*p*-Value		
	Intervention		Control		Intervention		Control				
	DL	NDL	DL	NDL	DL	NDL	DL	NDL			
	*Mean ± Standard Deviation*								Group*Time	Limb Dom*Time	Three-Way Interaction Effect
Variables Muscle Activity (% of Maximum)											
Average Biceps Femoris	0.12 ± 0.03	0.12 ± 0.05	0.10 ± 0.04	0.12 ± 0.04	0.10 ± 0.04	0.10 ± 0.07	0.10 ± 0.04	0.11 ± 0.04	0.569	0.624	0.981
Peak Biceps Femoris	0.58 ± 0.15	0.55 ± 0.18	0.48 ± 0.22	0.57 ± 0.17	0.50 ± 0.18	0.41 ± 0.22	0.44 ± 0.15	0.51 ± 0.15	0.389	0.528	0.699
Average Semitendinosus	0.13 ± 0.04	0.13 ± 0.04	0.13 ± 0.04	0.12 ± 0.05	0.10 ± 0.06	0.15 ± 0.06	0.11 ± 0.05	0.11 ± 0.05	0.540	0.080	0.334
Peak Semitendinosus	0.56 ± 0.17	0.50 ± 0.17	0.55 ± 0.16	0.55 ± 0.20	0.49 ± 0.28	0.55 ± 0.17	0.49 ± 0.15	0.50 ± 0.21	0.486	0.323	0.488
Average Vastus Lateralis	0.12 ± 0.04	0.12 ± 0.04	0.12 ± 0.06	0.11 ± 0.06	0.10 ± 0.04	0.11 ± 0.05	0.10 ± 0.05	0.08 ± 0.05	0.447	0.645	0.571
Peak Vastus Lateralis	0.50 ± 0.19	0.53 ± 0.13	0.51 ± 0.18	0.49 ± 0.22	0.45 ± 0.17	0.52 ± 0.24	0.47 ± 0.18	0.37 ± 0.20	0.505	0.823	0.353
Average Vastus Medialis	0.14 ± 0.04	0.13 ± 0.06	0.13 ± 0.04	0.12 ± 0.05	0.13 ± 0.04	0.13 ± 0.07	0.12 ± 0.05	0.09 ± 0.06	0.488	0.672	0.359
Peak Vastus Medialis	0.55 ± 0.12	0.49 ± 0.15	0.51 ± 0.14	0.53 ± 0.20	0.47 ± 0.18	0.44 ± 0.20	0.53 ± 0.18	0.38 ± 0.25	0.876	0.242	0.105

(DL) Dominant Limb (NDL) Non-Dominant Limb.

**Table 5:** Mean ± standard deviation (SD) values for normally distributed data are reported, along with results from a three-factor mixed model ANOVA for peak Biceps Femoris and Semitendinosus muscle activity during the load phase of the CODAT, performed with the dominant limb (DL) and non-dominant limb (NDL).

	PRE—Intervention				POST—Intervention				*p*-Value		
	Intervention		Control		Intervention		Control				
	DL	NDL	DL	NDL	DL	NDL	DL	NDL			
*Parametric Analysis*	*Mean ± Standard Deviation*								Group*Time	Limb Dom*Time	Interaction Effect
Variables Muscle Activity (% of Maximum)											
Peak Biceps Femoris	0.53 ± 0.14	0.51 ± 0.13	0.48 ± 0.19	0.58 ± 0.15	0.51 ± 0.19	0.37 ± 0.24	0.56 ± 0.13	0.48 ± 0.18	0.212	0.013 *	0.633
Peak Semitendinosus	0.51 ± 0.15	0.44 ± 0.14	0.45 ± 0.12	0.49 ± 0.21	0.30 ± 0.19	0.39 ± 0.14	0.47 ± 0.16	0.36 ± 0.15	0.173	0.945	0.006 *

* and bold Denotes significance. (DL) Dominant Limb/(NDL) Non-Dominant Limb.

Table 6 presents the descriptive statistics for non-normally distributed data, along with results from independent comparisons between groups and paired comparisons within groups, for average and peak muscle activity EMG during the load phase of the CODAT. Data are reported separately for the dominant limb (DL) and non-dominant limb (NDL) at pre- and post-intervention time points.

Independent groups analyses revealed significant post-intervention differences in average ST muscle activity in DL, and in average VM muscle activity in NDL. The intervention group exhibited lower average ST muscle activity in DL (*p* = 0.001) and higher average VM muscle activity in NDL (*p* = 0.041) compared to the control group. Notably, no differences were found between groups in any muscles at the pre-intervention time point. Therefore, the intervention group exhibited a significant reduction in average ST muscle activity from pre- to post-intervention in DL (*p* = 0.004), along with a significant increase in average VM muscle activity in both limbs (*p* < 0.001 for both limbs). In contrast, the control group demonstrated a significant decrease in average VM muscle activity from pre- to post-intervention in both limbs (*p* < 0.001 for both limbs) and a significant reduction in peak VM muscle activity from pre- to post-intervention in NDL (*p* = 0.026) (Table 6).

**Table 6:** Median and interquartile range [Q1–Q3] values for non-normally distributed data are presented, along with results from the Mann–Whitney U test between groups and with results from the Wilcoxon test within-group, for peak and average Biceps Femoris, Semitendinosus, Vastus Medialis, and Vastus Lateralis muscle activity during the load phase of the CODAT, performed with the dominant limb (DL) and non-dominant limb (NDL).

	PRE—Intervention								POST—Intervention								Wilcoxon *p*-Value (PRE—POST)		Effect Size d Cohen (PRE—POST)	
	Intervention		Control		Mann Whitney U		Effect Size d Cohen		Intervention		Control		Mann Whitney U		Effect Size d Cohen					
	DL	NDL	DL	NDL	DL	NDL	DL	NDL	DL	NDL	DL	NDL	DL	NDL	DL	NDL				
			*Median [Interquartile range [Q1, Q3]*														Intervention Related Samples Analyses	Control Related Samples Analyses	Intervention Related Samples Analyses	Control Related Samples Analyses
Variables Muscle Activity (% of Maximum)																				
Average Biceps Femoris	0.10 [0.08, 0.12]	0.10 [0.08, 0.12]	0.10 [0.08, 0.13]	0.12 [0.09, 0.15]	0.883	0.163	0.00	2.00 ^†^	0.10 [0.07, 0.13]	0.09 [0.03, 0.14]	0.11 [0.09, 0.13]	0.10 [0.07, 0.12]	0.424	0.318	1.00	2.00 ^†^	DL: 0.657 NDL: 0.156	DL: 0.156 NDL: 0.420	DL: 0.00 NDL: 2.00 ^†^	DL: 1.00 NDL: 2.00 ^†^
Average Semitendinosus	0.09 [0.06, 0.12]	0.09 [0.07, 0.11]	0.09 [0.08, 0.11]	0.11 [0.08, 0.13]	0.883	0.118	0.00	2.00 ^†^	0.05 [0.02, 0.08]	0.09 [0.05, 0.10]	0.09 [0.07, 0.12]	0.08 [0.06, 0.11]	0.001 *	0.782	5.00 ^†^	1.00	DL: 0.004 * NDL: 0.652	DL: 0.750 NDL: 0.117	DL: 4.00 ^†^ NDL: 0.00	DL: 1.00 NDL: 3.00 ^†^
Average Vastus Lateralis	0.15 [0.11, 0.17]	0.13 [0.11, 0.16]	0.12 [0.07, 0.17]	0.13 [0.08, 0.16]	0.303	0.335	2.00 ^†^	3.00 ^†^	0.14 [0.10, 0.16]	0.14 [0.09, 0.17]	0.13 [0.07, 0.16]	0.08 [0.06, 0.11]	0.351	0.173	2.00 ^†^	2.00 ^†^	DL: 1.000 NDL: 0.089	DL: 0.324 NDL: 0.128	DL: 1.00 NDL: 2.00 ^†^	DL: 1.00 NDL: 1.00
Peak Vastus Lateralis	0.66 [0.48, 0.74]	0.64 [0.61, 0.71]	0.59 [0.46, 0.68]	0.65 [0.51, 0.72]	0.219	0.660	1.11 ^†^	1.88 ^†^	0.68 [0.60, 0.74]	0.68 [0.39, 0.77]	0.58 [0.38, 0.68]	0.43 [0.32, 0.61]	0.083	0.207	1.99 ^†^	1.67 ^†^	DL: 0.756 NDL: 0.121	DL: 0.459 NDL: 0.124	DL: 0.00 NDL: 1.90 ^†^	DL: 1.00 NDL: 1.67 ^†^
Average Vastus Medialis	0.14 [0.11, 0.18]	0.14 [0.09, 0.17]	0.14 [0.10, 0.17]	0.12 [0.09, 0.17]	0.660	0.708	1.00	1.00	0.15 [0.11, 0.18]	0.16 [0.07, 0.19]	0.13 [0.09, 0.16]	0.10 [0.06, 0.14]	0.245	0.041 *	2.00 ^†^	2.55 ^†^	DL: 0.000 * NDL: 0.000 *	DL: 0.000 * NDL:0.000 *	DL: 1.10 ^†^ NDL: 1.26 ^†^	DL: 1.10 ^†^ NDL: 3.00 ^†^
Peak Vastus Medialis	0.62 [0.51, 0.73]	0.60 [0.41, 0.68]	0.60 [0.53, 0.69]	0.62 [0.48, 0.72]	0.613	0.386	0.28	0.66	0.66 [0.53, 0.70]	0.57 [0.43, 0.71]	0.64 [0.57, 0.72]	0.41 [0.29, 0.61]	0.684	0.126	0.50	2.00 ^†^	DL: 0.943 NDL: 0.538	DL: 0.381 NDL: 0.026 *	DL: 0.00 NDL: 0.39	DL: 0.85 NDL: 3.20 ^†^

* and bold Denotes significance/^†^ denotes effect size d > 1. (DL) Dominant Limb/(NDL) Non-Dominant Limb.

**Table 7:** Kinematics and muscle activity pairwise comparisons for significant limb dominance by time interactions (Limb Dominance*Time).

		Mean Difference	*p*-Value	Effect Size d Cohen	95% Confidence Intervals for Differences	
					Lower Bound	Upper Bound
**Variable—Kinematics**						
Thigh Extension Angular Velocity	DL_PRE and POST	−34.62	0.040 *	2.20 ^†^	−67.62	−1.62
	NDL_PRE and POST	−33.81	0.053	2.48 ^†^	−68.02	0.40
**Variable—Muscle Activity EMG**						
Peak Biceps Femoris Load Phase	DL_PRE and POST	−0.03	0.432	1.00	−0.10	0.05
	NDL_PRE and POST	0.12	0.003 *	3.79 ^†^	0.04	0.19

* and bold Denotes significance/^†^ denotes effect size d > 1. (PRE) Pre-intervention Assessment/(POST) Post-intervention Assessment/(DL) Dominant Limb. (NDL) Non-Dominant Limb.

**Table 8:** Kinematic and muscle activity pairwise comparison for significant three-way interactions (Group*Limb Dominance*Time).

			Mean Difference	*p*-Value	Effect Size d Cohen	95% Confidence Intervals for Differences	
						Lower Bound	Upper Bound
**Variables—Kinematics** ***ROM Thigh Sagittal Angular Velocity***							
Intervention	DL	PRE-POST	65.14	0.137	0.44	−23.07	153.34
	NDL	PRE-POST	−37.49	0.442	0.07	−138.36	63.39
Control	DL	PRE-POST	−10.65	0.834	0.27	−116.16	94.87
	NDL	PRE-POST	157.38	0.015 *	0.65	34.37	280.39
**Variable—Muscle Activity EMG** ***Peak Semitendinosus Load Phase***							
Intervention	DL	PRE-POST	0.21	0.006 *	1.23^†^	0.07	0.34
	NDL	PRE-POST	0.05	0.362	0.14	−0.06	0.16
Control	DL	PRE-POST	−0.02	0.677	0.36	−0.12	0.08
	NDL	PRE-POST	0.13	0.103	0.71	−0.03	0.28

* and bold Denotes significance/^†^ denotes effect size d > 1. (PRE) Pre-intervention Assessment/(POST) Post-intervention Assessment/(DL) Dominant Limb (NDL) Non-Dominant Limb/ROM (Range of Motion).

## 4. Discussion

The aim of this study was to examine the effects of a 12-week multicomponent periodized program on movement quality and motor control in female youth football players, based on changes in three-dimensional knee angular velocity and muscle activity of the hamstrings and quadriceps during a COD task. The primary findings revealed that the program significantly improved both kinematic and muscle activity patterns during COD tasks, supporting our hypothesis. Specifically, post-intervention, the intervention group increased sagittal plane shank angular velocity, reflecting optimized functional performance strategy; reduced shank and thigh valgus angular velocity in the coronal plane, and diminished thigh peaks and range of angular velocities in the transverse plane, which suggests the multicomponent program enhanced quality of movement in all three planes during the CODAT. Currently, muscular activity modifications characterized by reduced average and peak ST muscle activity, indicating less compensatory hamstring activation is required to stabilize the joint dynamically, alongside increased VM muscle activity, demonstrate a more efficient contribution of the medial thigh stabilizers of the knee relative to the lateral thigh muscles during the LOAD phase. Notably, most of the functional adaptations were observed in DL, which initially presented higher baseline values associated with the ACL injury mechanism.

In the sagittal plane, the intervention group demonstrated significantly higher post-intervention shank extension, flexion, and sagittal ROM angular velocities in both DL and NDL compared to the control group, following the multicomponent program. Notably, the intervention significantly increased shank flexion angular velocity in NDL, while the control group exhibited a significant decrease in thigh sagittal ROM angular velocity from pre- to post-intervention in NDL. These kinematic adaptations suggest that, following the multicomponent program, the intervention group demonstrated greater effectiveness in rapidly decelerating the initial flexion-directed angular velocity and subsequently generating the extension-directed angular velocity required for body reorientation during COD. This reflects an optimized transition from the flexion-oriented displacement to the propulsion phase, dominated by extension-oriented displacement [15,19,29,62]. Consequently, the intervention group may exhibit a more efficient kinematic strategy in this sagittal plane, consistent with enhanced performance in football-specific tasks, such as faster cutting execution or rapid knee extension, which appears to have developed as previously described in the literature [15,19,62,63]. Notably, these improvements occurred in both limbs, indicating a balanced effect of the intervention. Given that the ACL functions as the primary restraint against anterior tibial shear, providing approximately 70–87% of the resistance in the sagittal plane, these findings indicate that improvements in sagittal plane quality of movement may contribute to the reduction or reversal of potential biomechanical parameters associated with ACL injury mechanisms [11,32].

The intervention group significantly reduced thigh valgus angular velocity in NDL and a reversal of previously elevated shank valgus angular velocity in DL, suggesting improved coronal plane kinematic control post-intervention compared to the control group. Furthermore, the intervention group exhibited greater shank varus and increased coronal ROM angular velocities in DL. In the transverse plane, no significant between-group differences were observed after the program; however, within the intervention group, significant pre- to post-intervention reductions were found in thigh internal and external rotation angular velocities in DL, as well as in transverse ROM in both DL and NDL. Valgus collapse, defined by knee abduction and excessive transverse motion, is widely recognized as a primary risk factor for ACL injury [9,11,26,27]. In our study, the control group showed progression toward this pattern during the intervention period, possibly due to the seasonal physical load. The intervention group, particularly in DL, may mitigate the kinematics associated with the ACL injury mechanism with a more favorable kinematic pattern. While prior studies have reduced knee abduction [12,18,21], this program uniquely addressed combined dysfunctional kinematic adjustments in both the coronal and transverse planes, especially during the early load phase of COD, which aligns with high prevalence timing of ACL injury [33]. These findings support recent evidence indicating that increased angular velocity and ROM, rather than rotation direction alone, are the most critical clinical concerns [11,25,64].

Finally, no significant main effects or interactions were observed for muscle activity in the PREP phase of CODAT. However, in the LOAD phase, the intervention group exhibited a significant reduction in both average and peak ST muscle activity in DL, along with a significant increase in average VM in both limbs. Conversely, the control group showed a significant decline in average and peak VM muscle activity over the follow-up period. Considering the multiplanar knee kinematics involved during COD tasks, the hamstrings and quadriceps act as key stabilizers of the knee [28,35,65]. Specifically, ST compresses the medial compartment and synergistically works with VM to provide medial stability and counteract the pivot shift mechanism [25,36,37,38]. The improved movement quality previously described may reflect enhanced motor control. Zebis et al. reported that ACL-injured players, who adopted a more upright posture during COD, showed preferential ST muscle activity compared to other thigh muscles. In this context, the reduced hamstring activity observed in the intervention group may indicate improved neuromuscular efficiency, likely associated with optimized knee positioning during dynamic stabilization tasks [36,43,45]. Furthermore, recent findings by Jeong et al. demonstrated that significant improvements in coronal plane risk-related kinematic parameters were accompanied by increased VM muscle activity. Accordingly, the increased VM muscle activity suggests enhanced function of the medial knee stabilizers and improved lateral-to-medial muscle balance, a recognized protective factor against ACL injury [25,38]. In contrast, the control group decreases VM activation, which may be attributed to the cumulative effects of the seasonal physical loading.

### 4.1. Limitations

This study presents several limitations. First, this study employed a non-randomized controlled design; future studies using randomized allocation of players or teams could strengthen the external validity of the findings. Second, although multiple criteria were applied to quantify and monitor training load to ensure compliance with pre-planned values and comparable exposure across groups, standardizing the warm-up routines of the control group could further strengthen the baseline and improve the reliability of comparative analyses. Third, while the 12-week intervention demonstrated efficacy, prospective follow-up is necessary to evaluate long-term adaptations, the maintenance of effects in reducing biomechanical parameters associated with the ACL injury mechanism, and the potential risk of detraining. According to the literature, specific prevention programs can reduce ACL injury rates by up to 67% [17,18]; therefore, incorporating such measures could help verify preventive effects. Future research could also incorporate subjective measures to investigate the relationship between biomechanical improvements and the player’s perceived functional changes. While this study employed self-reported questionnaires to monitor load perception and training adequacy, including these outcomes in conjunction with objective biomechanical data may better inform clinical progression, optimize physical load management, and support individualized and sustainable implementation of ACL injury prevention strategies within football training environments.

### 4.2. Clinical Contributions

The implementation of a multicomponent program specifically designed to automate the COD functional mechanisms, based on neuromuscular training, COD technique modification, and real-time feedback, has proven to be an effective strategy, reliable even under high-intensity COD demands, and closely linked to ACL risk injury. The program structure, utilizing a mesocycle-based periodization model, incorporates progressively demanding objectives that provide objective criteria for progression, aligned with the player’s improvement capabilities. The session format, carried out in pairs and organized in a circuit, allows for continuous verbal feedback from coaches and peers, while also facilitating video recording for subsequent visual feedback. This dual-feedback system enhances self-perception, supports functional integration, and increases overall program effectiveness. Finally, in alignment with recent clinical guidelines, this approach holds strong clinical value for female youth football players, a group characterized by high neuromuscular plasticity. As such, early implementation may promote long-term improvements in movement quality and motor control, significantly contributing to the reduction in developing biomechanical dysfunctional adjustments associated with ACL injury mechanisms.

## 5. Conclusions

The 12-week multicomponent periodized program effectively modified three-dimensional knee kinematics and muscle activity during the COD task in female youth football players. Improvements in movement quality were reflected by increased shank extension, flexion, and sagittal ROM angular velocities, alongside reduced thigh and shank valgus and enhanced shank varus and coronal ROM angular velocities. Additionally, the intervention significantly reduced thigh internal and external rotation, lowering transverse ROM angular velocity. These kinematic adaptations were accompanied by improvements in motor control, evidenced by reduced average ST and peak hamstrings muscle activity, and increased VM activity. Functional changes were more pronounced in DL, potentially due to poorer baseline function. In summary, the proposed multicomponent periodized program based on neuromuscular training, COD technique modification, and real-time feedback proved effective in enhancing movement quality and motor control. These adaptations contribute to the reduction in potential biomechanical dysfunctional adjustments associated with ACL injury mechanisms, supporting the effectiveness of the program as a targeted injury-prevention strategy for female youth football players.

## Data Availability

The datasets presented in this study are available on request from the corresponding author. All data covered by this study are included in this manuscript.

## Abbreviations

The following abbreviations are used in this manuscript:

ACL	Anterior Cruciate Ligament
EMG	Electromyography
ST	Semitendinosus
BF	Biceps Femoris
VM	Vastus Medialis
VL	Vastus Lateralis
PREP	Preparation phase
LOAD	Load phase
ROM	Range of Motion
DL	Dominant Limb
NDL	Non-Dominant Limb
CODAT	Change of Direction and Acceleration Test
COD	Change of Direction
RPE	Rating of Perceived Exertion
